# A Hydroxypyrone-Based Inhibitor of Metalloproteinase-12 Displays Neuroprotective Properties in Both *Status Epilepticus* and Optic Nerve Crush Animal Models

**DOI:** 10.3390/ijms19082178

**Published:** 2018-07-25

**Authors:** Jonathan Vinet, Anna-Maria Costa, Manuel Salinas-Navarro, Giuseppina Leo, Lieve Moons, Lutgarde Arckens, Giuseppe Biagini

**Affiliations:** 1 Laboratory of Experimental Epileptology, Department of Biomedical, Metabolic and Neural Sciences, University of Modena and Reggio Emilia, 41125 Modena, Italy; jonathan.vinet@unimore.it (J.V.); annamaria.costa@unimore.it (A.-M.C.); giuseppina.leo@unimore.it (G.L.); 2 Laboratory of Neural Circuit Development and Regeneration, Department of Biology, KU Leuven, 3000 Leuven, Belgium; manuel.salinas@um.es (M.S.-N.); lieve.moons@kuleuven.be (L.M.); 3 Laboratory of Neuroplasticity and Neuroproteomics, Department of Biology, KU Leuven, 3000 Leuven, Belgium; lut.arckens@kuleuven.be; 4 Center for Neuroscience and Neurotechnology, University of Modena and Reggio Emilia, 41125 Modena, Italy

**Keywords:** metalloproteinase-12, *status epilepticus*, optical neuropathy, blood–brain barrier leakage, pilocarpine, retinal ganglion cells

## Abstract

Recently, we showed that matrix metalloproteinase-12 (MMP-12) is highly expressed in microglia and myeloid infiltrates, which are presumably involved in blood–brain barrier (BBB) leakage and subsequent neuronal cell death that follows *status epilepticus* (SE). Here, we assessed the effects of a hydroxypyrone-based inhibitor selective for MMP-12 in the pilocarpine-induced SE rat model to determine hippocampal cell survival. In the hippocampus of rats treated with pilocarpine, intra-hippocampal injections of the MMP-12 inhibitor protected *Cornu Ammonis 3* (CA3) and hilus of dentate gyrus neurons against cell death and limited the development of the ischemic-like lesion that typically develops in the CA3 *stratum lacunosum-moleculare* of the hippocampus. Furthermore, we showed that MMP-12 inhibition limited immunoglobulin G and albumin extravasation after SE, suggesting a reduction in BBB leakage. Finally, to rule out any possible involvement of seizure modulation in the neuroprotective effects of MMP-12 inhibition, neuroprotection was also observed in the retina of treated animals after optic nerve crush. Overall, these results support the hypothesis that MMP-12 inhibition can directly counteract neuronal cell death and that the specific hydroxypyrone-based inhibitor used in this study could be a potential therapeutic agent against neurological diseases/disorders characterized by an important inflammatory response and/or neuronal cell loss.

## 1. Introduction

Matrix metalloproteinases (MMPs) are endopeptidases whose expression is mainly triggered in pathological conditions involving inflammation [1]. MMP-12, also known as macrophage metalloelastase or macrophage elastase, is able to degrade a wide variety of extracellular matrix components, such as laminin, type IV collagen, fibronectin, chondroitin sulfate, and vitronectin [2,3]. By degrading basal membrane components, MMP-12 permits the entry of macrophages and other immune cells into injured tissues during inflammation [2]. In the case of the central nervous system (CNS), MMP-12 might be responsible for blood–brain barrier (BBB) disruption occurring in several neurological diseases/disorders characterized by inflammatory processes, such as spinal cord injury [4], multiple sclerosis [5], and ischemic or hemorrhagic strokes [6,7]. Therefore, clinical research on specific MMP-12 inhibitors has become a major therapeutic interest [3].

Temporal lobe epilepsy (TLE), the most common form of epilepsy in adult humans [8], is characterized by inflammatory processes consisting of activated microglia, reactive astrocytes, local expression of pro-inflammatory cytokines, BBB leakage, and peripheral immune cell infiltration, which are thought to play a central role in the initiation and maintenance of seizures, starting in the acute phase defined as the “initial precipitating injury”, which is represented by, *inter alia*, the *status epilepticus* (SE) [9,10]. Recent studies suggested that ictogenesis might be associated with high levels of MMP-9 in patients suffering of various types of epilepsy [11]. Since MMP-9 is also associated with neuronal death, aberrant synaptic plasticity and inflammation, all of them occurring during epileptogenesis [10], MMP-9 has become a potential therapeutic target in epilepsy [11,12,13]. Recently, we have observed that activated microglia and infiltrating macrophages not only express MMP-9, but also present high levels of MMP-12 starting 24 h after SE [14], suggesting a possible involvement of this MMP in epileptogenesis.

Here we used a hydroxypyrone-based inhibitor, which has been demonstrated to be highly selective for MMP-12 [15], to test whether specific MMP-12 inhibition may represent a putative drug against neuronal cell death occurring after SE. We show that inhibition of MMP-12 is neuroprotective in the CA3 and in the *hilus* of the dentate gyrus (dentate *hilus*, DH) regions of the hippocampus. It also limits the development of the typical ischemic-like lesion that appears in the CA3 *stratum lacunosum-moleculare* after SE [10]. Moreover, MMP-12 inhibition reduces immunoglobulin G (IgG) and serum albumin extravasation into brain parenchyma. Finally, to rule out any possible involvement of seizure modulation in the neuroprotective effects of MMP-12 inhibition, we also demonstrate neuroprotection of the MMP-12 inhibitor in a murine optic nerve crush (ONC) model, thus indicating that this property might apply to different neurological diseases/disorders characterized by an important inflammatory response and/or neuronal cell loss.

## 2. Results

### 2.1. The Hydroxypyrone-Based MMP-12 Inhibitor Protects Neurons in the CA3 and DH

**Figure 1:**
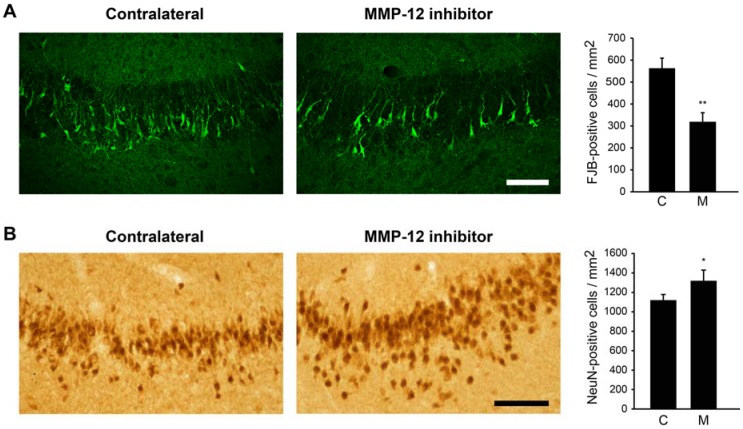
Effect of MMP-12 inhibition on neuronal cell death and survival in the CA3 hippocampal region of rats after pilocarpine-induced SE. Brain sections were stained for Fluoro-Jade B (FJB) (**A**) to evaluate neuronal cell death or against NeuN (**B**) to evaluate neuronal survival. The specific MMP-12 inhibitor significantly prevents the appearance of FJB-positive cells in the CA3 pyramidal layer compared to the contralateral hemisphere, while it increases neuronal survival, as illustrated by an increase in the number of NeuN-positive cells, indicating a neuroprotective effect. Quantification was performed using ImageJ. C = contralateral hemisphere, M = MMP-12 inhibitor. Data are shown as mean ± SEM. n = 7. * *p* < 0.05, ** *p* < 0.01; Student’s *t*-test. Scale bar: A = 50 µm, B = 100 µm.

Since MMP-12 degrades a wide variety of components of the BBB, we hypothesized that its inhibition could be beneficial in the context of damages occurring after SE. Rats that experienced SE were thus injected in one hippocampus twice/day for two days with a hydroxypyrone-based MMP-12 inhibitor, starting 24 h after SE. The contralateral hippocampus served as control. We first evaluated the neuronal cell death in the CA3 hippocampal region using Fluoro-Jade B (FJB), as a marker of apoptotic/necrotic neurons. We counted 560.33 ± 46.26 FJB-positive cells/mm^2^ in the contralateral hippocampus (Figure 1A). The inhibition of MMP-12 significantly reduced the number of neurons undergoing apoptosis/necrosis (*p* = 0.0017). Indeed, we counted 316.53 ± 43.51 FJB-positive cells/mm^2^ in the ipsilateral hippocampus (Figure 1A). These data were confirmed when looking at neuron-specific nuclear protein (NeuN) staining. We counted 1113.31 ± 60.24 NeuN-positive cells/mm^2^ in the whole CA3 *stratum pyramidalis* of the contralateral hippocampus while 1313.10 ± 111.28 NeuN-positive cells/mm^2^ were present in the same areas of the ipsilateral hippocampus (Figure 1B).

**Figure 2:**
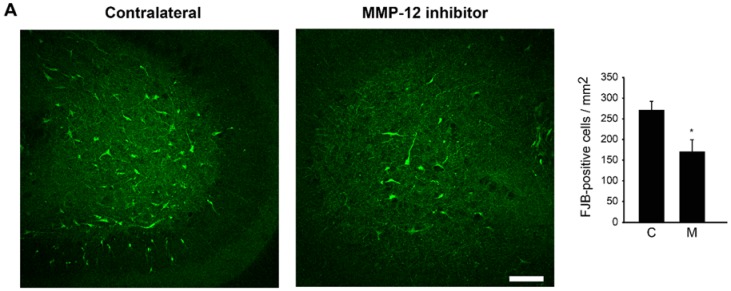

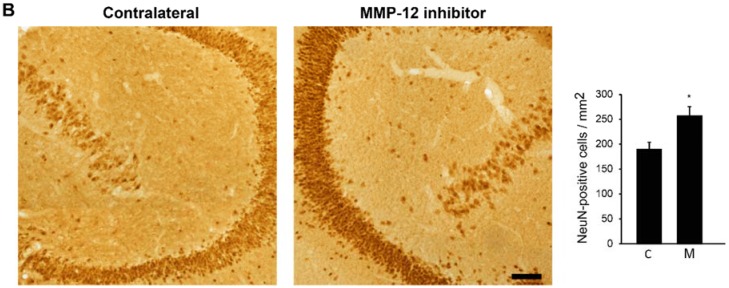
Effect of MMP-12 inhibition on neuronal cell death and survival in the dentate gyrus (DH) of rats after pilocarpine-induced SE. Brain sections were stained for FJB (**A**) to evaluate neuronal cell death or against NeuN (**B**) to evaluate neuronal survival. The specific MMP-12 inhibitor significantly prevents the appearance of FJB-positive cells in the DH compared to the contralateral hemisphere, while it increases neuronal survival, illustrated by an increase in the number of NeuN-positive cells, indicating a neuroprotective effect. Quantification was performed using ImageJ. C = contralateral hemisphere, M = MMP-12 inhibitor. Data are shown as mean ± SEM. n = 7. * *p* < 0.05; Student’s *t*-test. Scale bars: 100 µm.

A similar mode of protection was also observed in the DH of the hippocampus. Indeed, we counted 269.94 ± 21.37 FJB-positive cells/mm^2^ in the DH of the contralateral hippocampus compared to 167.18 ± 29.90 FJB-positive cells/mm^2^ in the ipsilateral DH (Figure 2A), which represents a significant reduction in neuronal cell death (*p* = 0.015). Again, this was also reflected when looking at NeuN staining, as we counted 189.43 ± 13.57 NeuN-positive cells/mm^2^ in the contralateral DH against 256.63 ± 19.04 NeuN-positive cells/mm^2^ in the ipsilateral DH (Figure 2B). Overall, these data strongly suggest that the inhibition of MMP-12 protects CA3 and DH neurons against neuronal cell death occurring after SE. This neuroprotection is not due to a direct effect of the inhibitor over microglia as no change in microglia activated morphology was observed between the ipsilateral and contralateral hippocampi (Figure S1).

### 2.2. Inhibition of MMP-12 Limits the Developement of the Ischemic-Like Lesion Present in the CA3 Stratum Lacunosum-Moleculare

**Figure 3:**
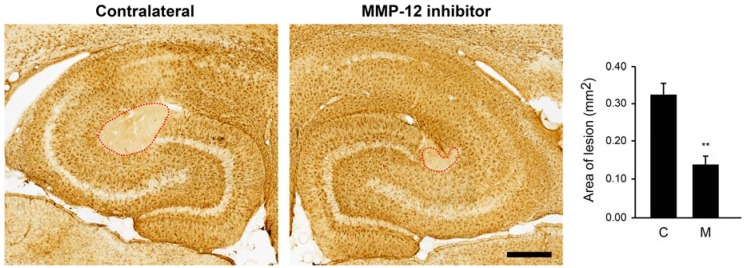
Effect of MMP-12 inhibition on the ischemic-like lesion in the CA3 *stratum lacunosum-moleculare* of rats after pilocarpine-induce SE. Brain sections were stained against GFAP, a marker of astrocytes, which are absent in the lesion occurring in the CA3 *stratum lacunosum-moleculare*. The selective MMP-12 inhibitor significantly prevented the enlargement of the lesion area. Quantification of the area (red-dotted circle) was performed using the image analysis software KS300. C = contralateral hemisphere, M = MMP-12 inhibitor. Data are shown as mean ± SEM. n = 7. ** *p* < 0.01; Student’s *t*-test. Scale bar: 500 µm.

We next investigated whether inhibition of MMP-12 could also have a protective effect on the ischemic-like lesion occurring after SE. Indeed, we have previously described that this lesion occurs in the CA3 *stratum lacunosum-moleculare* and then shrank after two weeks from SE [16,17,18]. This lesion can be easily observed by staining hippocampi against glial fibrillary acidic protein (GFAP), since astrocytes are completely absent from the lesion. Interestingly, inhibition of MMP-12 led to a significant reduction of the ischemic-like lesion area (Figure 3). More precisely, the mean lesion area in the contralateral hippocampus was 0.322 ± 0.032 mm^2^, while the MMP-12 inhibitor significantly reduced it to 0.138 ± 0.023 mm^2^ (*p* = 0.001).

### 2.3. The Hydroxypyrone-Based MMP-12 Inhibitor Reduces BBB Leakage at the Site of the Ischemic-Like Lesion

**Figure 4:**
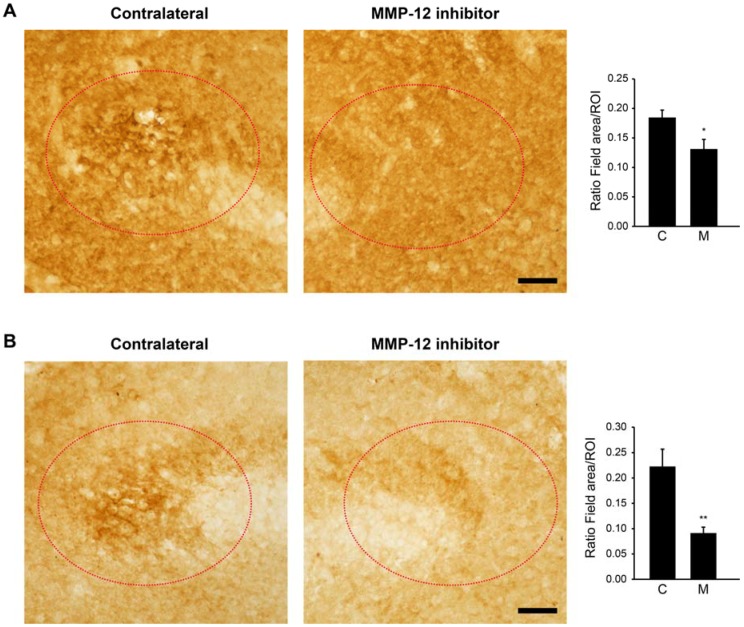
Effect of MMP-12 inhibition on IgG and albumin extravasation in the CA3 *stratum lacunosum-moleculare* of rats after pilocarpine-induced SE. Brain sections were stained against local IgG (**A**) and albumin (**B**). The specific MMP-12 inhibitor significantly decreased edema as revealed by IgG and albumin extravasation. Quantification of the field area inside the region of interest (red-dotted circle) was performed using the image analysis software KS300. C = contralateral hemisphere, M = MMP-12 inhibitor. Data are shown as mean ± SEM. n = 7. * *p* < 0.05, ** *p* < 0.01; Student’s *t*-test. Scale bars: 50 µm.

The decrease in the size of the ischemic-like lesion induced by the MMP-12 inhibitor could result from a reduced leakage of the BBB, which occurs normally with SE [19,20,21]. To examine this possibility, we evaluated the ingress of local IgG as well as serum albumin at the site of the ischemic-like lesion. Interestingly, inhibition of MMP-12 reduced the levels of IgG infiltrates at the site of the ischemic-like lesion (Figure 4A). Indeed, the field area of IgG staining was significantly diminished in the ipsilateral side (0.13 ± 0.016 µm^2^ vs. 0.183 ± 0.013 µm^2^; *p* = 0.024). Similar findings were obtained with serum albumin (Figure 4B). The field area of serum albumin was significantly reduced at the lesion site of the ipsilateral hemisphere (0.089 ± 0.011 µm^2^ vs. 0.221 ± 0.033 µm^2^; *p* = 0.003). Overall, these data indicate that inhibition of MMP-12 reduces the infiltration of local IgG and serum albumin, suggesting a reduced leakage of the BBB.

### 2.4. Zonula Occludens-1 (ZO-1) Is Another Marker for the Ischemic-Like Lesion

**Figure 5:**
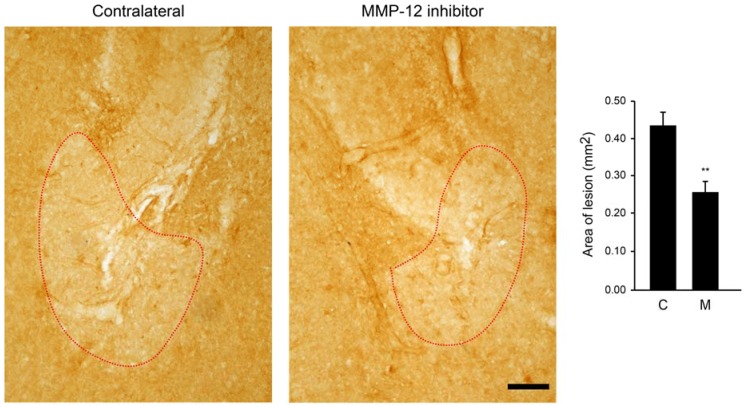
Effect of MMP-12 inhibition on Zonula Occludens-1 (ZO-1) labeling in the CA3 *stratum lacunosum-moleculare* of rats after pilocarpine-induced SE. Brain sections were stained again ZO-1, a marker of tight junctions. Interestingly, ZO-1 staining was absent in the ischemic lesion. The specific MMP-12 inhibitor induced a significant decrease of the lesion area (red-dotted circle). Quantification of the area was performed using the image analysis software KS300. C = contralateral hemisphere, M = MMP-12 inhibitor. Data are shown as mean ± SEM. *n* = 7. ** *p* < 0.01; Student’s *t*-test. Scale bar: 50 µm.

We next investigated if the reduced ingress of IgG and albumin at the site of the lesion could be linked to a diminution of the BBB leakage. We used ZO-1 as a marker of BBB integrity. Interestingly, the labeling of ZO-1 was approximately similar to the one of GFAP in that absence of ZO-1 staining delineated the area of the ischemic-like lesion. We thus quantified the lesion area and we observed a loss of staining similar to that found for GFAP in the contralateral hemisphere (Figure 5). More precisely, the lesion area was 0.43 ± 0.036 mm^2^. Inhibition of MMP-12 induced a significant reduction of the lesion area in the ipsilateral hemisphere (0.256 ± 0.03 mm^2^; *p* = 0.003), indicating that ZO-1 serves as an additional marker of the ischemic-like lesion and that the BBB integrity is somewhat better preserved in the treated area.

### 2.5. The Hydroxypyrone-Based MMP-12 Inhibitor Is also Neuroprotective in a Murine ONC Model

We have recorded epileptic activity/seizures following SE up to two days after pilocarpine-induced SE [22], an observation consistent with those made by other laboratories, reviewed in [23]. To rule out any possible involvement of seizure modulation in the neuroprotective effects of MMP-12 inhibition, we investigated whether MMP-12 contributes to retinal ganglion cell (RGC) survival after axonal damage induced by crush. Both vehicle- and inhibitor-treated mouse groups were subjected to unilateral ONC and to repeated intravitreal injections of vehicle or MMP-12 inhibitor, respectively.

**Figure 6:**
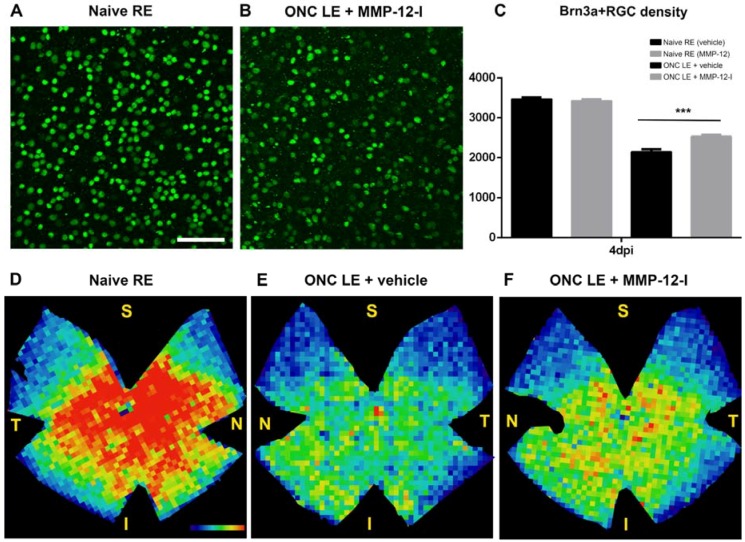
Effect of MMP-12 inhibition on retinal ganglion cell (RGC) survival and distribution after optic nerve crush (ONC). Immunohistochemical stainings for Brn3a on flat mounted retinas showing Brn3a + RGCs from a control (**A**) and an injured retina from the MMP-12 inhibitor-treated group 4 dpi (**B**). Both images illustrate the superotemporal medial retina. A decrease in the number of Brn3a + RGCs is observed after axonal lesion. (**C**) Bar graph of the Brn3+RGC density in whole mount retinas after ONC in vehicle and inhibitor-treated mice. At 4 dpi Brn3a + RGC survival is significantly higher in the MMP-12 inhibitor treated group compared to vehicle mice (*p* < 0.001) for the injured left eye, with normal RGC counts for the normal right eye of both groups. Representative isodensity maps, generated by assigning a color according to the RGC density value, illustrate Brn3a + RGC from flat-mounted control, (**D**) and experimental retinas from vehicle (**E**) and treated (**F**) mice, and reveal the decreased number of surviving Brn3a + RGCs at 4 dpi. The control retina shows a normal density, with highest (red) density in the central and lowest (blue) within the peripheral retina. RGC loss after ONC was diffuse and affected the whole retina. A substantially stronger decrease in RGC density is observed in the vehicle only group retinas as compared to retinas of the inhibitor treated group. S, superior; N, nasal; T, temporal; I, inferior. Data are shown as mean ± SEM. *n* = 6–8. *** *p* < 0.001; Bonferroni post-hoc test. Scale bar: 50 µm.

Upon ONC, the number of brain-specific homeobox/POU domain protein 3A (Brn3a) + RGCs both in vehicle-and inhibitor-treated mice decreased progressively over time post-lesion, with a Brn3a + RGC survival rate in vehicle mice of approximately 62% at four days post injury (dpi), which is similar to previous reported studies [24,25,26]. However, MMP-12 inhibitor-treated mice showed a Brn3a + RGC survival of 74% at the same time post ONC (Figure 6A–C), clearly indicating that the number of Brn3a + RGCs was significantly higher in the MMP-12 inhibitor treated group compared to the vehicle control group, at 4 dpi (2.535 ± 35 vs. 2.149 ± 61; *p* < 0.001) (Figure 6C). These data suggest that the inhibition of a functional MMP-12 protein is neuroprotective at early time points after ONC.

The spatial distribution of surviving Brn3a + RGCs in both groups is illustrated by isodensity maps (Figure 6D–F). Control retinas in both groups showed a similar distribution of Brn3a + RGC (Figure 6D) with a horizontal region in the superior retina near the optic disk with highest RGC density, as previously described [27]. Isodensity maps of the injured retinas of both groups showed a diffuse Brn3a + RGCs loss affecting the entire retina, without any focal or sectorial loss. Injured treated retinas at 4 dpi (Figure 6F) revealed warmer colors than those of vehicle group mice (Figure 6E), indicating increased RGC survival at this time point.

In summary, both, quantitative analysis and distribution of the surviving Brn3a + RGCs indicated that MMP-12 inhibition induces a neuroprotective effect on axotomized RGCs at four days after axonal crush injury.

## 3. Discussion

MMPs are expressed in the CNS mainly during inflammation and, if uncontrolled, they often mediate adverse effects [28]. Thus, controlling their expression or inhibiting their activity is of therapeutic interest. TLE is characterized by inflammatory processes, which have been observed and described both in humans and animal models [9,29]. Moreover, glutamate released during seizures induces an increase in MMP-2 and MMP-9 protein expression and activity levels, resulting in BBB leakage [30]. In this regard, high levels of MMP-9 have been found in patients suffering of various types of epilepsy [11] and Fragile X syndrome, in which many patients show high susceptibility to epilepsy [31]. Since high levels of MMP-9 are associated with neuronal death, aberrant synaptic plasticity, and inflammation during epileptogenesis, MMP-9 has become a potential therapeutic target in epilepsy.

We recently reported that microglia and infiltrating macrophages express not only MMP-9 during early epileptogenesis but also high levels of MMP-12 [14]. Here, we demonstrate that inhibition of MMP-12 is able to protect hippocampal neurons against cell death occurring after SE, as well as to reduce the ischemic-like lesion that is observed in the CA3 *stratum lacunosum-moleculare* of the hippocampus. How this neuroprotection occurs remains unclear. A possible mechanism could be that by inhibiting the enzymatic activity of MMP-12, the damage to the BBB is decreased.

BBB dysfunction in epilepsy has been demonstrated by several groups [19,20,21]. This leads to the infiltration of blood cells as well as molecules from the periphery inside the CNS, enhancing the inflammatory response and brain injury. Indeed, it was proposed that the ingress of albumin in the CNS is responsible for triggering a gliotic injury response by astrocytes [19,20,21]. Our data indicate that selective inhibition of MMP-12 reduces the ingress of IgG and albumin from the periphery, corroborating the hypothesis of reduced BBB dysfunction. A decreased leakage from the BBB could prevent the infiltration of peripheral blood-born molecules and immune cells and thus, decrease the inflammatory milieu that is in part responsible for the neuronal cell death occurring during epileptogenesis [32,33,34,35,36,37]. Further studies will need to be performed to verify the effect of MMP-12 inhibition on the BBB properties as well as on the number of infiltrating blood cells and the expression of inflammatory cytokines/chemokines in epileptogenesis.

Recently, it was demonstrated that the expression and localization of tight junction proteins which are responsible for the BBB integrity, such as ZO-1, are altered after SE, and that these changes were mediated by MMP-9 activity [38]. Here, we showed that ZO-1 staining is absent in the ischemic lesion and that the area of the lesion is reduced after MMP-12 inhibition, suggesting an attenuation of ZO-1 degradation. MMP-12 is able to activate other MMPs such as pro-MMP-2 and pro-MMP-3, which in turn can activate pro-MMP-1 and pro-MMP-9 [39]. It is thus possible that the specific MMP-12 inhibitor could indirectly attenuate the activity of MMP-9, leading to a protection of tight junctions proteins. Further studies are needed to investigate if this molecular mechanism is occurring.

Abnormal expression of MMP-12 has been related to several CNS diseases [40]. MMP-12 expression is highly upregulated in neurons and glial cells following peripheral nerve injury [41], brain ischemic stroke [7,8,42], spinal cord injury [4], epilepsy [43] and multiple sclerosis [5,44]. Interestingly, increased MMP-12 expression has also been reported after ONC [45], in retinal detachment [46] and ischemic retinopathy [47] models. All of these previous findings indicate that an abnormal overexpression of MMP-12 may contribute to neuronal degeneration in various optic retinopathies [46,47]. Therefore, to rule out any possible involvement of seizure modulation in the neuroprotective effects of MMP-12 inhibition, we also aimed at investigating the potential role of MMP-12 in neuronal survival processes in the retina, which for now remain not well understood.

Here, we demonstrate that specific inhibition of MMP-12 is capable to protect RGCs against cell death occurring after ONC. Previous in vivo experiments also showed that chronic treatment with MMP-12 inhibitor ameliorated Npc1 deficiency-induced axonal pathology in the striatum [48]. In the same line, MMP-12 deficiency protected against spinal cord injury, a neuroprotective effect suggested to relate to a decreased permeability of the blood-spinal barrier and reduced microglial activation and macrophage infiltration in MMP-12 null mice [4]. Also, specific knockdown of MMP-12 after focal cerebral ischemia offered neuroprotection. This could be mediated via a reduced MMP-9 activation, with MMP-12 being able to induce MMP-9, as well as via the reduction of protein expression of apoptotic molecules that are downstream of tumor necrosis factor α (TNFα) signaling, since TNFα protein expression was reduced upon MMP-12 knockdown [7,8]. Similarly, a cannabinoid receptor 2 agonist was shown to prevent thrombin-induced BBB damage by inhibiting microglia activation and by downregulating MMP-9 and MMP-12 expression [49]. Finally, knockdown of HuR, an RNA-binding protein that regulates gene expression, attenuated microglia migration/invasion in mutant SOD1 mice, due to down-regulation of MMP-12 expression [50]. Although the exact mechanism by which MMP-12 inhibition results in neuroprotection in both the pilocarpine and ONC models remains unknown, it could be due to one or a combination of the mechanisms described above: blood-barrier disruption, MMP9 activation and/or control over TNFα. Our results indicate that abnormal expression of MMP-12 may contribute to neuronal degeneration in TLE and ONC, and that inhibition of MMP-12 activity or expression may represent a therapeutic approach for TLE and optical neuropathy.

In conclusion, we provide evidence that specific inhibition of MMP-12 prevents neuronal cell death and reduces the ischemic-like lesion that occur in the hippocampus after SE. The neuroprotective property of this inhibitor could also be observed in the ONC model, indicating that it was independent of any possible modulatory property of MMP-12 inhibition of seizures induced by pilocarpine. This finding suggests that MMP-12 inhibition could be a potential therapeutic target to counteract inflammatory-induced neuronal cell death. This neuroprotection may affect positively the occurrence of spontaneous recurrent seizures and prevent the development of chronic epilepsy.

## 4. Materials and Methods

### 4.1. Status Epilepticus Model

#### 4.1.1. Animals

Eight male Sprague Dawley rats of eight weeks of age (Charles River Laboratories, Calco, Italy) were housed in standard Makrolon^®^ (Bayer, Milan, Italy) cages in a quiet environment with ad libitum access to water and food. All experiments were performed according to the European Council Directive of 22 September 2010 (2010/63/EU) and approved by the Italian Ministry of Health (799/2017-PR).

#### 4.1.2. Cannulae Implantation

After deep anesthesia with isoflurane (Vetefarma Srl, Madonna dell’Olmo, Itlay), the skin was shaved, disinfected (10% povidone-iodine, Betadine^®^ skin solution, Meda Pharma, Milan, Italy), and opened to expose the scalp. Guiding holes were drilled and 4 mm guide cannulae (C315G, Bilaney Consultant GmbH, Dusseldorf, Germany) were implanted in the right hippocampal CA3 *radiatum* (bregma–5 mm, 5 mm lateral from midline) and cemented on the rat head. To reduce pain and risk of infection, a gel containing 2.5 g lidocaine chloride, 0.5 g neomycin sulfate and 0.025 g fluocinolone acetonide (Neuflan^®^ gel, Molteni Farmaceutici, Milan, Italy) was applied after surgery. Rats were kept under a heat lamp and monitored until complete recovery from anesthesia. Animals were left to recover for one week. One rat was removed from the study since the cannula was not implanted correctly.

#### 4.1.3. *Status Epilepticus* Induction

Rats received an intraperitoneal (i.p.) injection of *N*-methylscopolamine (Sigma-Aldrich, Milan, Italy; 1 mg/kg) to prevent the peripheral effects of cholinergic stimulation. 30 min later, rats received an injection of pilocarpine (Sigma-Aldrich, 380 mg/kg, i.p.) to induce SE. Rats were visually monitored for seizures until SE developed. Diazepam (Hospira Italia, Naples, Italy; 20 mg/kg) was used to quell seizure activity after a SE of 10 min [16,51]. In all animals, drug-induced responses were observed directly and graded according to a modification of the Racine’s scale [52]. Briefly, we considered: (i) non-convulsive seizures, ranked as stage 1 to stage 3; (ii) convulsive seizures, ranked as stage 4 to stage 5; (iii) SE (stage 6), considered as the stage in which rats either did not recover normal behaviour (i.e., exploration, grooming or motor reaction to stimuli) between one seizure and the other, or in which they displayed continuous shaking for more than 5 min [51,53].

#### 4.1.4. MMP-12 Inhibitor Injection

The hydroxypyrone-based MMP-12 inhibitor was prepared as previously published [15]. 24 h after SE, rats were anaesthetized with isoflurane and placed on a stereotaxic apparatus equipped with a micro-injector (Quintessential Stereotaxic Injector, Stoelting C.O., Dublin, Ireland). Animals received 1 µL of the MMP-12 inhibitor (2 mM in 10% dimethyl sulfoxide (DMSO, Sigma-Aldrich), in PBS) through the cannula. More precisely, MMP-12 inhibitor was injected in the right hippocampal CA3 *radiatum*, while the left hippocampal CA3 *radiatum* served as an internal control. The duration of the injection was 5 min. The cannula was left in place for another 2 min to prevent spill-over. After the injection, the cannula was removed, and animals were left to recover from the anesthesia. These procedures were repeated twice/day for two days.

#### 4.1.5. Immunohistochemistry

24 h after the last injection (72 h after SE), rats anaesthetized with isoflurane were transcardiacally perfused with phosphate buffered saline (PBS, pH 7.4) followed by Zamboni’s fixative (pH 6.9). Brains were extracted, post-fixed at 4 °C in the same fixative for 24 h, cryoprotected in 15% and 30% sucrose solutions, and stored at −80 °C until used. Horizontal sections of 50 μm were cut using a freezing sliding microtome (Leica SM2000R, Nussloch, Germany). Sections were washed three times in PBS, treated with 3% H_2_O_2_ in PBS (20 min), and blocked for 1 h with 5% normal goat serum (NGS, Sigma-Aldrich) in PBS containing 0.1% TritonX-100 (PBS-T). Sections were incubated overnight at 4 °C in PBS-T containing 1% NGS with the following antibody: mouse anti-NeuN (#MAB377 clone A60, 1:200, Millipore, Milan, Italy), mouse anti-GFAP (#G3893, 1:500, Sigma Aldrich), rabbit anti-albumin (#A000102-2, 1:5000, DAKO (Agilent Technologies), Cernusco sul Naviglio, Italy), rabbit anti-zonula occludens-1 (ZO-1, #61-7300, 1:200, TermoFisher Scientific, Monza, Italy), rabbit anti-ionized calcium-binding adapter molecule 1 (Iba1, #019-19741, 1:1000, Wako (DBA), Segrate, Italy). The next day, sections were incubated for 1 h with secondary antibodies (biotinylated goat anti-rabbit, biotinylated horse anti-mouse; all 1:200, Vectastain ABC-Peroxidase kits, DBA). For IgG immunostaining, sections were blocked in PBS-T 0.1% and then incubated for 1 h with an anti-rat IgG antibody (#LRPN1005V/AA, 1:200, Amersham Pharmacia Biotech, Cologno Monzese, Italy). All sections were then treated with the avidin-biotin-peroxidase complex (Vectastain ABC-Peroxidase kits, DBA). The immunostaining was performed in 0.05% 3,3-diaminobenzidine tetrahydrochloride for 5 min (DAB, Sigma-Aldrich) and developed by adding 0.03% H_2_O_2_. Finally, sections were washed in PBS, mounted on gelatin-coated slides and cover slipped with Eukitt^®^ (O. Kindler GmbH & Co, Freiburg, Germany).

#### 4.1.6. Fluoro-Jade B

Brain sections were mounted on gelatin-coated slides and dried at room temperature. Slides were immersed for 5 min into a solution containing 1% sodium hydroxide in 80% ethanol, washed for 2 min in 70% ethanol followed by 2 min in distilled water, before being oxidized in 0.06% potassium permanganate for 15 min. Sections were then stained for 15 min in a 0.004% solution of FJB (Millipore) diluted in 0.1% acetic acid. Slides were rinsed in deionized water for 3 min, dried on a pre-warmed hotplate at 50 °C, then cleared in xylene and cover-slipped with Eukitt^®^. Images were acquired using a Leica SP2 AOBS confocal microscope.

#### 4.1.7. Image Analysis

DAB-immunostained sections were evaluated with a Nikon Eclipse CiL (Nikon Instruments, Florence, Italy) equipped with 2×, 4× and 10× objectives and for each area of interest, images were digitally captured by a Nikon DS-Fi3 digital camera. The number of NeuN immunoreactive cells and, respectively, that of FJB-positive cells per mm^2^, were quantified and analyzed using ImageJ software (Fiji, version 1.52b). IgG and albumin immunoreactivity intensities were analyzed using the image analysis software KS300 (Carl Zeiss Vision GmbH, Munchen, Germany), as previously detailed [17,18,54,55,56], and expressed as the ratio of field area value over the ROI area. ROI was kept similar in all sections analyzed. Field areas, representing the total area covered by the specific immunoreactivity, were obtained after having measured background grey values in the angular bundle, which was faintly stained for the absence of specific objects. Then, threshold was manually adjusted for all the analyzed sections by subtracting a defined and constant value from the peak grey-level histogram, thus discriminating the specific objects from background. The image analysis software KS300 was also used to manually trace the unstained area upon GFAP and ZO-1 detection in the CA3 *stratum lacunosum-moleculare*. All measurements were from 4 sections taken in the dorso-ventral axis of the hippocampus and results are expressed as means and standard error of the mean (SEM).

### 4.2. ONC Model

#### 4.2.1. Animal Handling and Ethics Statement

All experiments were performed using 8–12 weeks old C57Bl/6J wild-type (WT, in total 20 animals obtained from Charles River Laboratories) mice of both sexes. All animals were housed under standard laboratory conditions according to a normal day/night rhythm with *ad libitum* access to food and water. For anaesthesia, a mixture of xylazine (10 mg/kg body weight) and ketamine (70 mg/kg body weight, Anesketin) was i.p. administered. Additionally, for surgery and intravitreal (ivt) injections, a drop of local analgesic, oxybuprocaine hydrochloride (0.4%, Unicaïne, Théa, Wetteren, Belgium) was applied to the treated eye. During recovery from anaesthesia, an ocular ointment (Tobrex; Alcon, Puurs, Belgium) was applied on the cornea to prevent corneal desiccation. All animals were sacrificed with an i.p. injection of an overdose of sodium pentobarbital (30 mg/kg, Nembutal).

All animal experiments were approved by the Institutional Ethical Committee of KU Leuven (P260/2014) and followed the guidelines of the Association for Research in Vision and Ophthalmology and the European Communities Council Directive of 22 September 2010 (2010/63/EU).

#### 4.2.2. Animal Surgery: Intraorbital ONC and Ivt Injection

Intraorbital ONC was induced in the left experimental eye, while the right eye served as an internal control, using previously established methods [24,25,57]. Briefly, the optic nerve was accessed by an incision in the conjunctiva temporally around the eye. The left optic nerve was crushed at 1 mm from the optic disk for 0.5 s, applying a constant and consistent force using cross-action forceps. Before and after the ONC procedure, funduscopy was performed to ensure a normal retinal perfusion. Ivt injection of 1 µL of the MMP-12 inhibitor at concentrations of 2 mM (10% DMSO in PBS), were performed at the day of surgery and 2 days post ONC (dpi) in the treated group. The vehicle control group received ivt injections of 1 μL vehicle only (10% DMSO in PBS) at similar time points. Ivt injections were performed using a glass capillary with a 50–70 μm outer diameter, connected to a Micro4 Microsyringe controller (World Precision Instruments, Freidberg, Germany), at a rate of 500 nL/s. Animals were sacrificed at 4 dpi.

#### 4.2.3. Immunohistochemistry

To determine retinal ganglion cell (RGC) survival, immmunofluorescent staining for Brn3a, a POU-domain transcription factor and reliable marker for RGCs [58,59] was performed on whole-mount retinas, flattened following established methods [24,27]. All retinas were permeated in PBS-T 0.5% by freezing them at −70 °C for 15 min, rinsed in PBS-T 0.5% and incubated overnight with primary antibody: polyclonal goat anti-Brn3a (sc-31984, 1:500, Santa Cruz, Heidelberg, Germany) in blocking buffer (PBS-T 2% containing 2% primary immune rabbit serum). Afterward, retinas were incubated for 2 h with Alexa-568-conjugated rabbit anti-goat secondary antibody (Invitrogen, Aalst, Belgium), washed in PBS and finally mounted with the vitreal side up on slides, and covered with Mowiol mounting medium.

#### 4.2.4. Image Analysis

Retinas were examined and photographed using a confocal laser confocal scanning microscope (FV1000-D, Olympus Corporation, Berchem, Antwerp, Belgium), controlled by Olympus Fluoview 4.2 software. Retinal whole-mount reconstructions were obtained by combining the different frames captured contiguously side-by-side in a raster scan pattern, with a 20× objective. A Z-stack of each frame of the entire RGC layer (20–30 planes, 3 μm interval) was created and a maximum intensity projection image was generated. All frames of a retina were combined automatically into a single image of the whole retina. Automated cell counting and isodensity maps were done using the in-house developed ImageJ script, as previously described [60].

### 4.3. Statistical Analysis

Data are represented as means with SEM. For the SE model, statistical comparison between groups was performed using a Student’s *t*-test. Statistical analyses were performed with SigmaPlot 13 (Systat Software, San Josè, CA, USA). For the ONC model, statistical analysis was done using Student’s *t*-test or one-way ANOVA with post-hoc Bonferroni multiple comparisons test, employing the Prism 6 software package (GraphPad Software, San Diego, CA, USA). Differences were considered statistically significant for *p* < 0.05.

## Supplementary Materials

Supplementary File 1

## Abbreviations

BBB	Blood-brain barrier
Brn3a	Brain-specific hemeobox/POU domain protein 3A
CA3	Cornu Ammonis 3
CNS	Central nervous system
DAB	3,3-Diaminobenzidine tetrahydrochloride
DH	Hilus of the dentate gyrus
DMSO	Dimethyl sulfoxide
FJB	Fluoro-Jade B
GFAP	Glial fibrillary acidic protein
IgG	Immunoglobulin G
Ivt	Intravitreal
MMP	Metalloproteinase
NeuN	Neuron-specific nuclear protein
ONC	Optic nerve crush
PBS	Phosphate buffer saline
RGC	Retinal ganglion cell
SE	Status epilepticus
SOD	Superoxide dismutase
TLE	Temporal lobe epilepsy
TNFα	Tumor necrosis factor α
ZO-1	Zonula occludens-1
