# Supplementary figures and images for: A Hydroxypyrone-Based Inhibitor of Metalloproteinase-12 Displays Neuroprotective Properties in Both Status Epilepticus and Optic Nerve Crush Animal Models
Source: Int J Mol Sci. 2018 Jul 25;19(8):2178. doi: 10.3390/ijms19082178 (PMC6121268; doi:10.3390/ijms19082178)

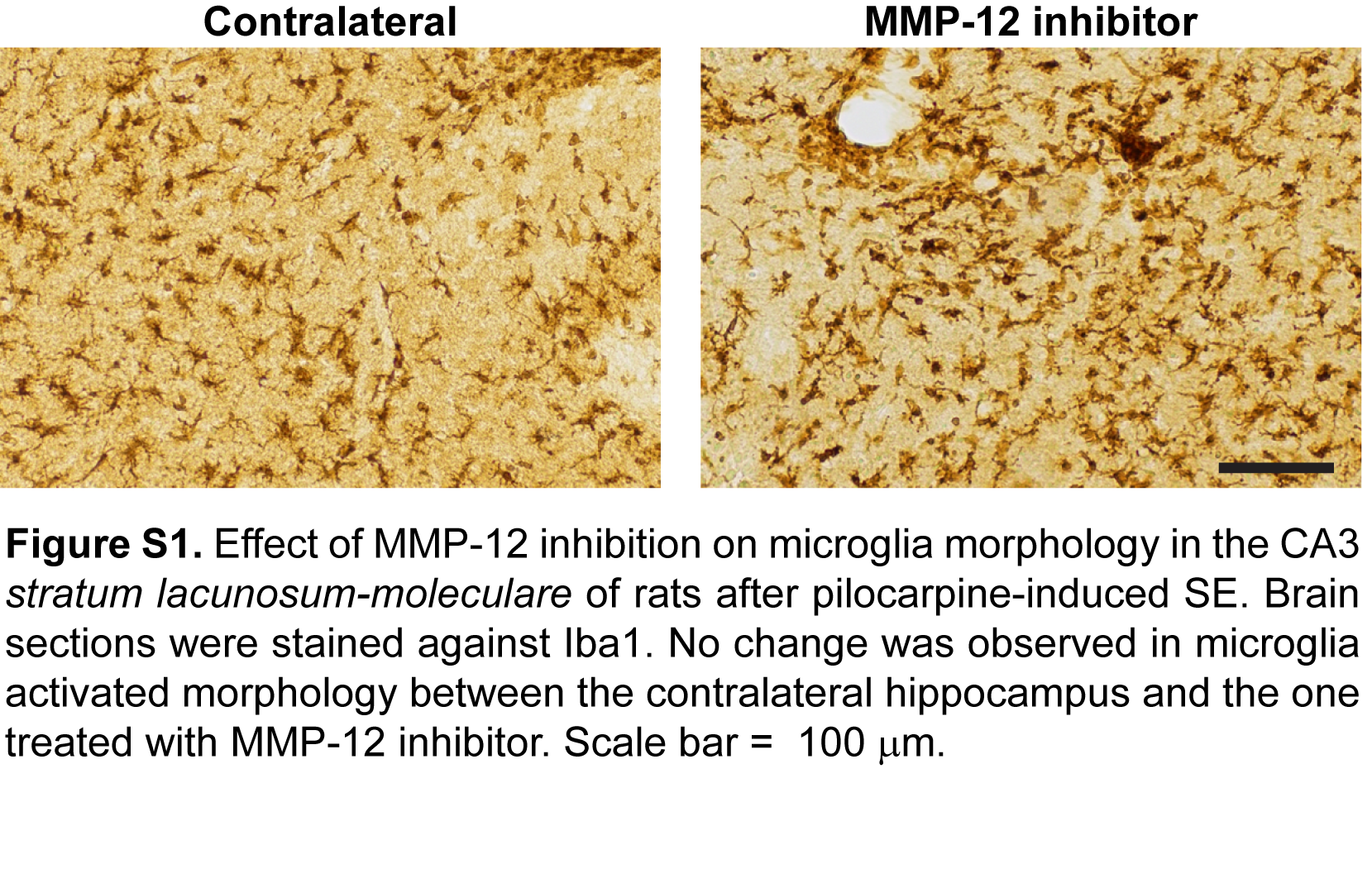

Supplement: Supplementary File 1 [file ijms-19-02178-s001.zip › ijms-328471-supplementary.tif]
